# Nilotinib first-line therapy in patients with Philadelphia chromosome-negative/BCR-ABL-positive chronic myeloid leukemia in chronic phase: ENEST1st sub-analysis

**DOI:** 10.1007/s00432-017-2359-9

**Published:** 2017-02-21

**Authors:** Andreas Hochhaus, Franҫois-Xavier Mahon, Philipp le Coutre, Ljubomir Petrov, Jeroen J. W. M. Janssen, Nicholas C. P. Cross, Delphine Rea, Fausto Castagnetti, Andrzej Hellmann, Gianantonio Rosti, Norbert Gattermann, Maria Liz Paciello Coronel, Maria Asuncion Echeveste Gutierrez, Valentin Garcia-Gutierrez, Beatrice Vincenzi, Luca Dezzani, Francis J. Giles

**Affiliations:** 1 Abteilung Hämatologie/Onkologie, Klinik für Innere Medizin II, Universitätsklinikum Jena, Am Klinikum 1, 07740 Jena, Germany; 2 Laboratoire Hématopoïèse Leucémique et Cible Thérapeutique, Université Victor Ségalen, Bordeaux, France; 3 Charité-Universitätsmedizin Berlin Campus Virchow, Berlin, Germany; 4 Ion Chiricuta Institute of Oncology, Cluj-Napoca, Romania; 5 Department of Hematology, VU University Medical Center, Amsterdam, The Netherlands; 6 Faculty of Medicine, University of Southampton, Southampton, UK; 7 Adult Hematology Department, Hôpital Saint-Louis, APHP, Paris, France; 8 Department of Experimental, Diagnostic and Specialty Medicine, Institute of Hematology “L. & A. Seràgnoli”, “S Orsola-Malpighi” University Hospital, University of Bologna, Bologna, Italy; 9 Department of Hematology, Medical University of Gdańsk, Gdańsk, Poland; 10 Department of Hematology, Oncology, and Clinical Immunology, Universitätsklinikum Düsseldorf, Düsseldorf, Germany; 11 Servicio de Hematología y Hemoterapia, IRYCIS, Hospital Universitario Ramón y Cajal, Madrid, Spain; 12 Hospital de Donostia, San Sebastian, Spain; 13 Novartis Oncology Region Europe, Origgio, Italy; 14 Division of Hematology Oncology, Developmental Therapeutics Program, Northwestern University Feinberg School of Medicine, Chicago, IL USA

**Keywords:** ENEST1st, Nilotinib, Chronic myeloid leukemia, Philadelphia chromosome negative/BCR-ABL positive

## Abstract

**Purpose:**

The ENEST1st sub-analysis presents data based on Philadelphia chromosome (Ph) status, i.e., Ph+ and Ph−/*BCR-ABL1* + chronic myeloid leukemia.

**Methods:**

Patients received nilotinib 300 mg twice daily, up to 24 months.

**Results:**

At screening, 983 patients were identified as Ph+ and 30 patients as Ph−/*BCR-ABL* + based on cytogenetic and RT-PCR assessment; 76 patients had unknown karyotype (excluded from this sub-analysis). In the Ph−/*BCR-ABL1* + subgroup, no additional chromosomal aberrations were reported. In the Ph+ subgroup, 952 patients had safety and molecular assessments. In the Ph−/*BCR-ABL1* + subgroup, 30 patients had safety assessments and 28 were followed up for molecular assessments. At 18 months, the molecular response (MR) 4 rate [MR^4^; BCR-ABL1 ≤0.01% on International Scale (IS)] was similar in the Ph−/*BCR-ABL1*+ (39.3%) and Ph+ subgroups (38.1%). By 24 months, the cumulative rates of major molecular response (BCR-ABL1^IS^ ≤0.1%;), MR^4^, and MR^4.5^ (BCR-ABL1^IS^ ≤0.0032%) were 85.7, 60.7, and 50.0%, respectively, in the Ph−/*BCR-ABL1* + subgroup, and 80.3, 54.7, and 38.3%, respectively, in the Ph+ subgroup. In both Ph−/*BCR-ABL1* + and Ph+ subgroups, rash (20 and 22%), pruritus (16.7 and 16.7%), nasopharyngitis (13.3 and 10.4%), fatigue (10 and 14.2%), headache (10 and 15.8%), and nausea (6.7 vs 11.4%) were frequent non-hematologic adverse events, whereas hypophosphatemia (23.3 and 6.8%), anemia (10 and 6.5%), and thrombocytopenia (3.3 and 10.2%) were the common hematologic/biochemical laboratory events.

**Conclusion:**

Based on similar molecular response and safety results in both subgroups, we conclude that Ph−/*BCR-ABL1* + patients benefit from nilotinib in the same way as Ph+ patients.

## Introduction

Chronic myeloid leukemia (CML) is characterized by the presence of Philadelphia (Ph) chromosome in >95% of the cases. The Ph chromosome, formed as a result of a reciprocal translocation between chromosomes 9 and 22, carries a region that expresses the chimeric *BCR-ABL1* gene which encodes for the BCR-ABL1 fusion protein (Bartram et al. 1983; de Klein et al. 1982; Rowley 1973; Shtivelman et al. 1987).

In very few patients (~5%) with CML, the Ph chromosome is not detectable despite *BCR-ABL1* positivity by fluorescent in situ hybridization or reverse transcriptase polymerase chain reaction (RT-PCR). The explanation for these cases are that there is a double recombination event involving chromosomes 9 and 22, and in some cases one or more other chromosomes (Bartram 1985; Fitzgerald and Morris 1991; Heim et al. 1985; La Starza et al. 2002; Nishigaki et al. 1992; Seong et al. 1999; Sessarego et al. 2000; Todoric-Zivanovic et al. 2006). Usually, patients with Ph-negative (Ph−)/*BCR-ABL1*-positive (*BCR-ABL1*+) CML are clinically not distinguishable from patients with Ph+ CML (Baccarani et al. 2013; Martiat et al. 1991; Seong et al. 1999).

Nilotinib, a second-generation BCR-ABL tyrosine kinase inhibitor (TKI) is approved for the treatment of adult patients with newly diagnosed Ph+ CML in chronic phase (CP) (Tasigna 2015). In the pivotal phase 3 ENESTnd trial in patients with newly diagnosed CML, nilotinib 300 mg twice daily demonstrated efficacy, with patients achieving early and deep molecular responses and consistent long-term safety profile (Hochhaus et al. 2016b; Kantarjian et al. 2011; Larson et al. 2012; Saglio et al. 2010). Nilotinib or other TKIs have not been systematically investigated in patients with Ph−/*BCR-ABL1* + CML.

The ENEST1st study evaluated the safety and efficacy of nilotinib 300 mg twice daily in a large population of patients with newly diagnosed Ph+ or Ph−/*BCR-ABL1* + CML-CP. In the overall population, the primary endpoint of molecular response (MR) 4 [MR^4^; BCR-ABL1 ≤0.01% on the International Scale (IS)] at 18 months was achieved by 38.4% of patients (Hochhaus et al. 2016a). Here, we present data from a sub-analysis of the ENEST1st study based on the Ph status, i.e., Ph+ CML and Ph−/*BCR-ABL1* + CML.

## Patients and methods

### Study design, patients and dosing

The European phase 3b, multicenter, single-arm, open-label ENEST1st trial enrolled adult patients (aged ≥18 years) with newly diagnosed (≤6 months) Ph+ or Ph−/*BCR-ABL1* + CML-CP, with molecular confirmation of the *BCR-ABL* fusion. Patients were required to have World Health Organization performance status ≤2. Detailed eligibility criteria were previously reported (Hochhaus et al. 2016a). Patients were treated with nilotinib 300 mg twice daily, and followed for up to 24 months. Dose escalation was not permitted, whereas dose interruptions were recommended in patients who experienced study drug-related, clinically significant nonhematologic or noncardiac adverse events (AEs) of grade 2/3 severity, or study drug-related white blood cell- or platelet-related events of grade 3/4 severity.

The primary endpoint of the study was the rate of MR^4^ at 18 months. The secondary endpoints included the rates of major molecular response (MMR; BCR-ABL1^IS^ ≤0.1%), MR^4^, and MR^4.5^ (BCR-ABL1^IS^ ≤0.0032%) at and by 12 and 24 months of treatment, and safety. This subanalysis presents data based on the Ph status at diagnosis (Hochhaus et al. 2016a).

### Assessments and definitions

Bone marrow cytogenetic assessments were performed within 8 weeks before the first dose of nilotinib. Cytogenetic assessments were performed and analyzed locally using standard methods on at least 20 metaphases; fluorescence in situ hybridization analyses were not used for response assessment. Patients with Ph−/*BCR-ABL1* + CML and those with unconfirmed Ph status at screening and no Ph+ metaphases at later time points were not assessed for cytogenetic responses.

At baseline, the *BCR-ABL1* transcript type was determined by multiplex polymerase chain reaction (PCR) (Cross et al. 1994) and DNA sequencing. In subsequent samples, *BCR-ABL1* transcripts were quantified every 3 months by quantitative real-time reverse transcriptase qRT-PCR testing of peripheral blood. Samples were analyzed at the designated European Treatment and Outcome Study (EUTOS) reference laboratories. For each sample, the ratio of *BCR-ABL1* transcripts vs control gene (*ABL*) transcripts converted to IS was calculated (Hughes and Branford 2006; Müller et al. 2008).

Molecular response was defined according to the definitions of EUTOS (Cross et al. 2012).

National Cancer Institute Common Terminology Criteria for Adverse Events version 4.0 was used for toxicity and adverse event reporting (NCI-CTCAE Version 4.0 2009).

### Statistical analyses

The subset of patients with major *BCR-ABL1* transcripts (i.e., b2a2 and/or b3a2) and ≤3 months of prior imatinib treatment were included in the molecular analysis population. Patients with minor *BCR-ABL1* transcripts were excluded as the standard qRT-PCR methodology was not optimized for the detection of minor *BCR-ABL1* transcripts.

The landmark analysis included patients with major *BCR-ABL1* transcripts, with no prior imatinib exposure and evaluable qRT-PCR assessments at 3 months. Patients who already achieved the target response of MMR, MR^4^, and MR^4.5^ at 3 months were excluded from the landmark analysis of MMR, MR^4^, and MR^4.5^, respectively.

To calculate response rates “at” a designated time point, patients were considered responders only if an assessment at that time point showed achievement of response. Response rates “by” a designated time point were calculated as cumulative response rates, counting all patients with a response detected at or before the specified time point as responders. All response rates were calculated as raw proportions.

### Ethics

This study was conducted in accordance with the International Conference on Harmonization Harmonized Tripartite Guidelines for Good Clinical Practice, the Declaration of Helsinki, and applicable local regulations. Informed consent was obtained from all individual participants included in the study. The protocol and informed consent forms were reviewed and approved by an institutional review board, independent ethics committee, or research ethics board before the study started at each participating institution. ENEST1st was registered in the EU Clinical Trials Registry (2009-017775-19) and ClinicalTrials.gov (NCT01061177).

## Results

### Patient disposition and characteristics

**Fig. 1:**
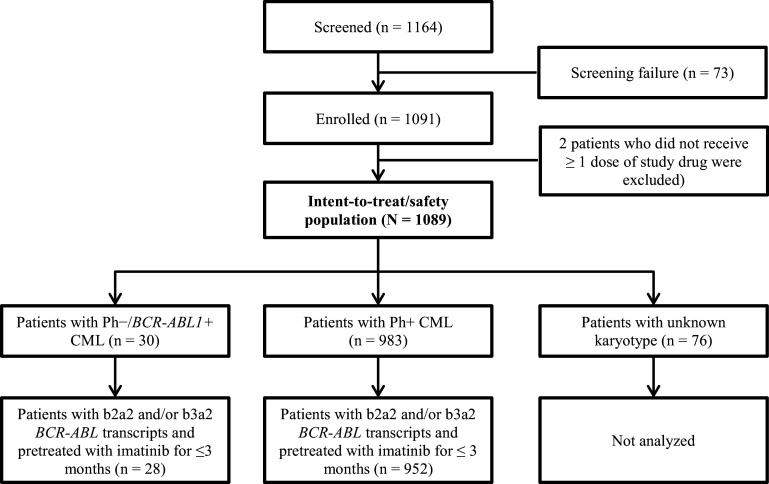
Patient disposition

The study enrolled 1091 patients from 2010 to 2012 across 307 sites in 26 European countries, and 1089 patients who received ≥1 dose of nilotinib 300 mg twice daily were evaluated. Based on cytogenetic assessment, 983 patients were identified as Ph+ and 30 patients were identified as Ph− at screening; 76 patients had unknown karyotype (Fig. 1). The 30 patients with Ph− status were positive for *BCR-ABL1* based on RT-PCR assessment. In the Ph− subset, no additional chromosomal aberrations were reported.

In the Ph+ subgroup, 952 patients were evaluable for safety and efficacy, and in the Ph−/*BCR-ABL1* + subgroup, 28 patients were evaluable for efficacy and all 30 patients were evaluable for safety (Fig. 1). Median age of patients with Ph−/*BCR-ABL1* + CML was 51.5 years (range 21.0–75.0). In total, 26 patients (86.7%) had low-risk EUTOS scores and 2 patients (6.7%) had high-risk EUROS scores. Low, intermediate, and high Sokal risk scores were detected in 10 (33.3%), 9 (30.0%), and 7 patients (23.3%), respectively. In the Ph+ CML subgroup, the median age was 53.0 years (range 18.0–91.0), EUTOS score was low in 806 patients (82.0%) and high in 90 patients (9.2%), and Sokal risk score was low, intermediate, and high in 342 (34.8%), 366 (37.2%), and 178 patients (18.1%), respectively (Table 1).

**Table 1:** Baseline characteristics and demographics

Parameter	Ph−/*BCR-ABL1* + CML (*n* = 30)	Ph+ CML (*n* = 983)
Age, median (range), years	51.5 (21.0–75.0)	53.0 (18.0–91.0)
Sex (Male/Female), *n* (%)	17 (56.7)/13 (43.3)	581 (59.1)/402 (40.9)
Race, *n* (%)		
Caucasian	30 (100)	941 (95.7)
Prior therapies^a,b^ *n* (%)		
None	5 (16.7)	301 (30.6)
Imatinib ≤1 month	2 (6.7)	59 (6.0)
Imatinib >1–2 months	3 (10.0)	62 (6.3)
Imatinib >2 months	10 (33.3)	35 (3.6)
Hydroxyurea	10 (33.3)	524 (53.3)
Type of BCR-ABL transcripts, *n* (%)		
b3a2	16 (53.3)	482 (49.0)
b2a2	10 (33.3)	362 (36.8)
b3a2 and b2a2	3 (10.0)	111 (11.3)
Not assessed at baseline	1 (3.3)	3 (0.3)
Sokal score, median (range)	0.87 (0.51–8.92)	0.86 (0.44–5.55)
High risk, *n* (%)	7 (23.3)	178 (18.1)
Intermediate risk, n (%)	9 (30.0)	366 (37.2)
Low risk, *n* (%)	10 (33.3)	342 (34.8)
Missing, *n* (%)	4 (13.3)	97 (9.9)
EUTOS score, median (range)	0.39 (0–0.94)	0.34 (0–3)
High risk, *n* (%)	2 (6.7)	90 (9.2)
Low risk, *n* (%)	26 (86.7)	806 (82.0)
Missing, *n* (%)	2 (6.7)	87 (8.9)

^a^Patients who received imatinib and hydroxyurea and/or other drugs are counted within the imatinib categories only. Patients who received hydroxyurea plus other drugs (not imatinib) are counted within the hydroxyurea category only

^b^Two additional patients in the Ph+ subgroup received therapies other than imatinib and/or hydroxyurea: one patient received cytarabine for 7 days, and other patient received capecitabine and oxaliplatin

### Molecular response

**Fig. 2:**
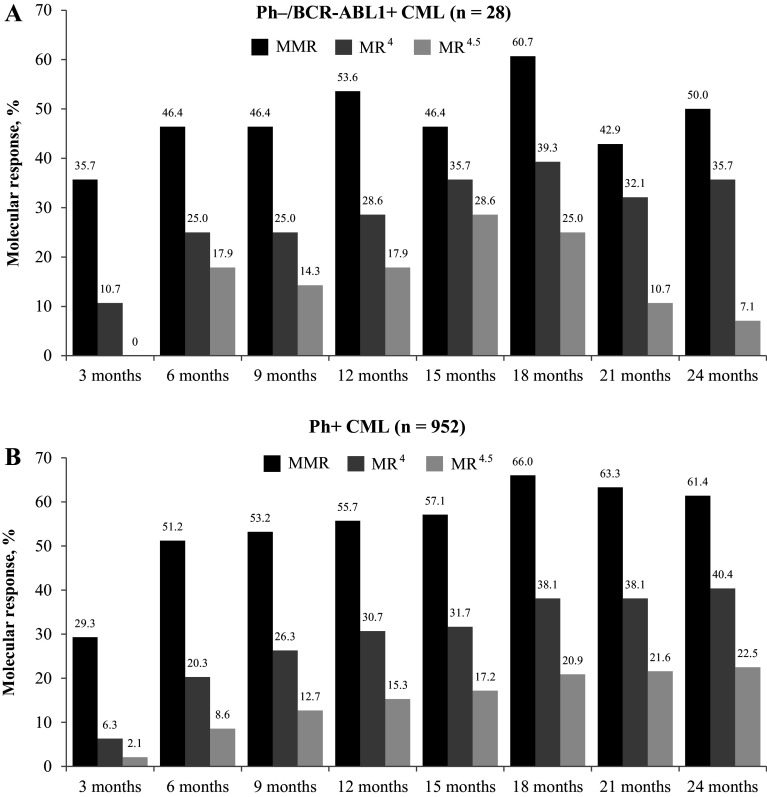
Molecular responses during treatment at different time points in Ph–/*BCR-ABL1* + CML (*n* = 28) (**a**) and Ph+ CML (*n * = 952) (**b**).* MMR* major molecular response (BCR-ABL1^IS^ ≤ 0.1%),* MR* molecular response,* MR* ^4^ MR with 4-log reduction in BCR-ABL transcript (BCR-ABL1^IS^ ≤ 0.01%),* MR* ^4.5^ MR with 4.5-log reduction in BCR-ABL transcript (BCR-ABL1^IS^ ≤ 0.0032%)

**Fig. 3:**
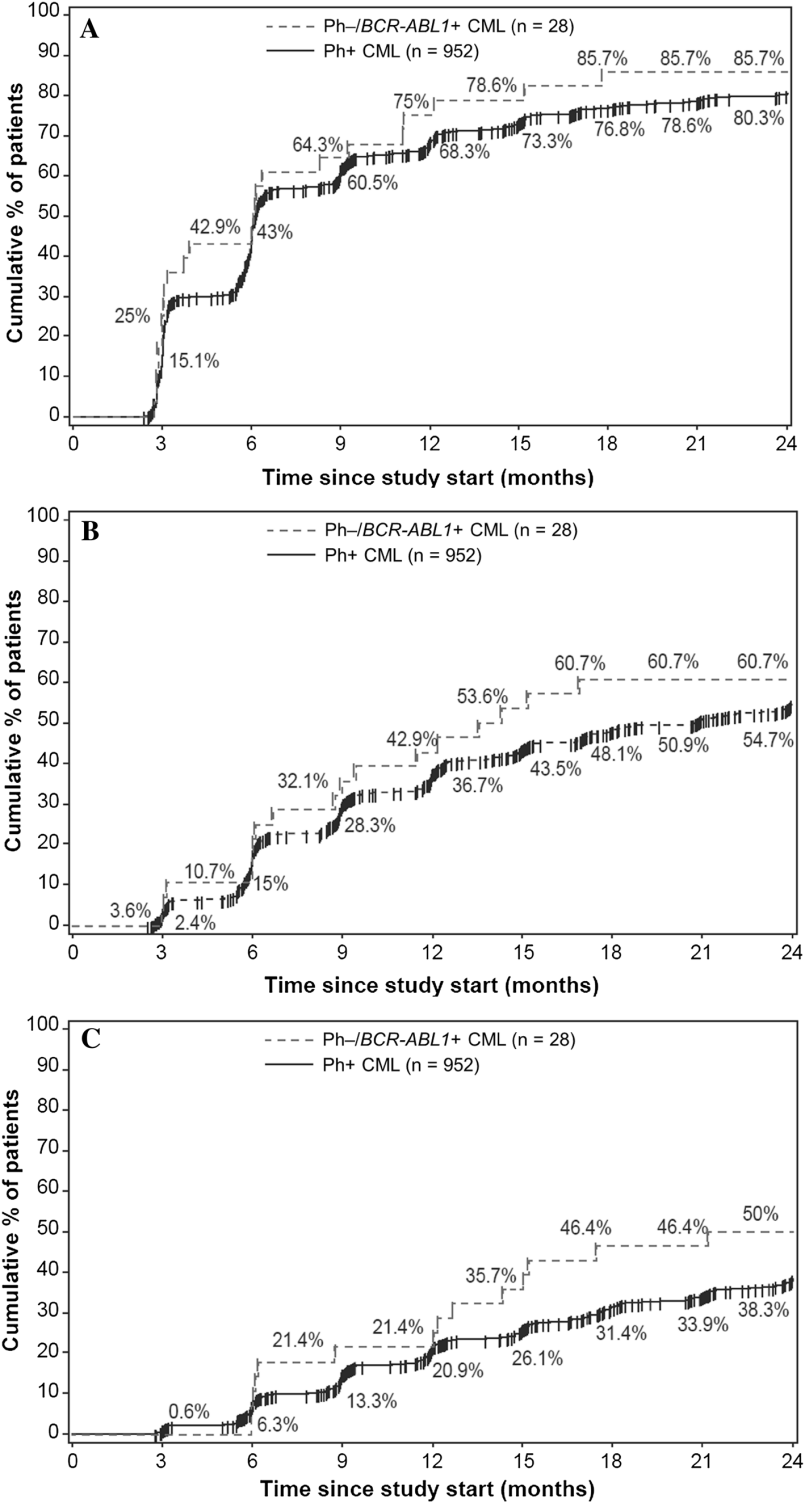
Cumulative rate of MMR **(a)**, MR^4^ **(b)**, and MR^4.5^ **(c)** in Ph–/*BCR-ABL1* + and Ph+ subgroups by 24 months. *MMR* major molecular response (BCR-ABL1^IS^ ≤0.1%), *MR* molecular response, *MR* ^*4*^ MR with 4-log reduction in BCR-ABL transcript (BCR-ABL1^IS^ ≤0.01%), *MR* ^*4.5*^ MR with 4.5-log reduction in BCR-ABL transcript (BCR-ABL1^IS^ ≤0.0032%)

In total, 28 patients met the criteria for molecular response analysis in the Ph−/*BCR-ABL1* + subgroup. The proportion of patients who achieved the primary endpoint of MR^4^ at 18 months was similar in both subgroups, with 39.3% (*n* = 11) in the Ph−/*BCR-ABL1* + subgroup and 38.1% (*n* = 363) in the Ph+ subgroup (Fig. 2). In the Ph−/*BCR-ABL1* + population, the MR^4^ rate was 28.6% at 12 months and 35.7% at 24 months. At 12 and 24 months, the respective rates of MMR were 53.6 and 50.0%, and that of MR^4.5^ were 17.9 and 7.1% (Fig. 2). By 12 months, the cumulative rate of MMR was 75.0%, MR^4^ was 42.9%, and MR^4.5^ was 21.4%. The cumulative rates of MMR, MR^4^, and MR^4.5^ by 24 months were 85.7, 60.7, and 50.0%, respectively (Fig. 3). In the Ph+ CML population, at 12 and 24 months, the rates of MMR were 55.7 and 61.4%, respectively, MR^4^ were 30.7 and 40.4%, respectively, and that of MR^4.5^ were 15.3 and 22.5%, respectively (Fig. 2). The cumulative rates of MMR, MR^4^, and MR^4.5^ were 68.3, 36.7, and 20.9% by 12 months, respectively, and 80.3, 54.7, and 38.3% by 24 months, respectively (Fig. 3).

### Landmark analysis

Of the 14 patients with Ph−/*BCR-ABL1* + CML, 12 (85.7%) had BCR-ABL1^IS^ ≤1%, and 1 had BCR-ABL1^IS^ >1 to ≤10% at 3 months. In patients with BCR-ABL1^IS^ ≤1% at 3 months, the cumulative incidence of MMR was 77.8% (7/9 patients), MR^4^ was 58.3% (7/12 patients), and MR^4.5^ was 41.7% (5/12 patients) by 24 months.

In the Ph+ CML subgroup, the cumulative rates of MMR by 24 months were 88% (300/338 patients), 57.4% (78/136 patients), and 36.4% (8/22 patients) in patients with BCR-ABL1^IS^ ≤1%, >1 to ≤10%, and >10% at 3 months, respectively. The cumulative rates of MR^4^ and MR^4.5^ by 24 months were 64.8% (333/514 patients) and 46.1% (251/545 patients), respectively, in patients with BCR-ABL1^IS^ >0% to ≤1% at 3 months, and 24.3% (33/136 patients) and 14% (19/136 patients), respectively, in patients with BCR-ABL1^IS^ >1 to ≤10% at 3 months.

### Safety

The most frequently reported (≥10%) nonhematological AEs (all grades) included rash (20.0%), pruritus (16.7%), nasopharyngitis (13.3%), diarrhea (10.0%), fatigue (10.0%), arthralgia (10.0%), headache (10.0%), and hypertension (10.0%) in the Ph−/*BCR-ABL1* + subgroup, and rash (22.0%), pruritus (16.7%), headache (15.8%), fatigue (14.2%), nausea (11.4%), alopecia (11.0%), and nasopharyngitis (10.4%) in the Ph+ subgroup (Table 2).

**Table 2:** Adverse events and laboratory abnormalities occurring in ≥ 10% of patients at any grade or ≥ 1% of patients at grade 3/4 in the Ph−/*BCR-ABL1* + CML or Ph+ CML subgroups

Patients, * n * (%)^a^	Ph−/*BCR-ABL1* + CML *n* = 30			Ph+ CML *n* = 952		
	All grades	Grade 3	Grade 4	All grades	Grade 3	Grade 4
Non-hematological events						
Rash	6 (20.0)	0	0	209 (22.0)	4 (0.4)	0
Pruritus	5 (16.7)	1 (3.3)	0	159 (16.7)	2 (0.2)	0
Nasopharyngitis	4 (13.3)	0	0	99 (10.4)	0	0
Diarrhea	3 (10.0)	0	0	86 (9.0)	1 (0.1)	0
Fatigue	3 (10.0)	0	0	135 (14.2)	7 (0.7)	0
Arthralgia	3 (10.0)	0	0	87 (9.1)	2 (0.2)	0
Headache	3 (10.0)	0	0	150 (15.8)	7 (0.7)	0
Hypertension	3 (10.0)	0	0	56 (5.9)	11 (1.2)	0
Dry skin	2 (6.7)	0	0	88 (9.2)	0	0
Nausea	2 (6.7)	0	0	109 (11.4)	5 (0.5)	0
Back pain	2 (6.7)	0	0	69 (7.2)	4 (0.4)	0
Myalgia	2 (6.7)	0	0	87 (9.1)	2 (0.2)	0
Urticaria	1 (3.3)	1 (3.3)	0	11 (1.2)	1 (0.1)	0
Drug hypersensitivity	1 (3.3)	1 (3.3)	0	1 (0.1)	0	0
Dermal cyst	1 (3.3)	1 (3.3)	0	3 (0.3)	0	0
Alopecia	1 (3.3)	0	0	105 (11.0)	1 (0.1)	0
Hematological laboratory events						
Anemia	3 (10.0)	0	3 (10.0)	0	0	62 (6.5)
Thrombocytopenia	1 (3.3)	0	1 (3.3)	0	1 (3.3)	97 (10.2)
Neutropenia	0	0	0	0	0	41 (4.3)
Biochemical laboratory events						
Hypophosphatemia	7 (23.3)	2 (6.7)	7 (23.3)	2 (6.7)	0	65 (6.8)
Alanine aminotransferase increase	4 (13.3)	1 (3.3)	4 (13.3)	1 (3.3)	0	79 (8.3)
Bilirubin increase	4 (13.3)	0	4 (13.3)	0	0	70 (7.4)
Lipase increase	3 (10.0)	0	3 (10.0)	0	0	71 (7.5)
Aspartate aminotransferase increase	3 (10.0)	0	3 (10.0)	0	0	45 (4.7)

^a^Excludes events that started >28 days after last dose of study drug or month 24

The most common (≥10%) hematological/biochemical laboratory abnormalities (all grades) were hypophosphatemia (23.3%), alanine aminotransferase increase (ALT, 13.3%), bilirubin increase (13.3%), aspartate aminotransferase increase (AST; 10.0%), lipase increase (10.0%), and anemia (10.0%) in the Ph−/*BCR-ABL1* + subgroup, and thrombocytopenia (10.2%) in the Ph+ subgroup (Table 2).

In patients with Ph−/*BCR-ABL1* + CML, two patients (7.1%) experienced grade 3 hypophosphatemia and one patient each (3.6%) experienced dermal cyst, pruritus, urticaria, and ALT increase of grade 3 severity; three patients experienced cardiovascular events, including four grade 3 events. Grade 4 anemia occurred in 1 patient (Table 2).

In the Ph+ subgroup, thrombocytopenia, lipase increase, neutropenia and hypophosphatemia, anemia, ALT increase, and bilirubin increase of grade 3 severity were experienced by 37 (3.9%), 30 (3.2%), 20 (2.1%), 19 (2%), 17 (1.8%), 14 (1.5%), and 13 (1.4%) patients, respectively. Grade 4 thrombocytopenia, neutropenia, and lipase increase were reported in 22 (2.3%), 8 (0.8%), and 7 (0.7%) patients, respectively (Table 2).

## Discussion

The development of BCR-ABL TKIs has revolutionized the therapeutic landscape of CML-CP. However, these TKIs have only been approved for the treatment of patients with Ph+ CML (Baccarani et al. 2013; Bisen and Claxton 2013; NCCN 2016). Sufficient literature is available on the effect of TKIs on patients with Ph+/*BCR-ABL1* + CML; however, the effect of TKIs in patients with Ph−/*BCR-ABL1* + CML has not been widely explored. This report, to the best of our knowledge, is the first of its kind to present data on the effect of nilotinib in patients with Ph−/*BCR-ABL1* + CML.

In a previous study, the efficacy of interferon-alpha was evaluated in patients with Ph−/*BCR-ABL1* + CML in early CP. Of the 14 patients who received interferon-alpha, 12 achieved complete hematologic remission, and the median survival duration was 60 months (range 3–>90 months). Patients with Ph+ CML and Ph−/*BCR-ABL1* + CML were found to have similar characteristics and outcomes (Cortes et al. 1995).

In the ENEST1st study, among 1052 patients evaluable, the cumulative rates of MMR, MR^4^, and MR^4.5^ were 80.4%, 55.2%, and 38.6%, respectively, by 24 months (Hochhaus et al. 2016a). At 24 months, the estimated overall survival rate was 98.9% (95% CI, 98.0–99.4%), with 13 on study deaths reported, and the estimated rate of freedom from progression to accelerated phase/blast crisis (AP/BC) was 99.4% (95% CI, 98.7–99.7%). None of the six patients who progressed to AP/BC on treatment died during study (Hochhaus et al. 2016a). Results from this study confirm that patients on nilotinib can achieve deep molecular responses, as previously seen in the ENESTnd study (Hochhaus et al. 2016b).

In the current sub-analysis of the ENEST1st trial, the primary endpoint of MR^4^ at 18 months was similar between Ph+ (39.3%) and Ph−/*BCR-ABL1*+ (38.1%) populations. The safety profile of nilotinib was also similar between the two populations, with the most frequently reported AEs being hypophosphatemia (23.3%), rash (20.0%), and pruritus (16.7%) in the Ph−/*BCR-ABL1* + population, and rash (22.0%), pruritus (16.7%), and headache (15.8%) in the Ph+ population. The overall safety results from this study were consistent with the safety profile of nilotinib and similar to that observed in the ENESTnd study (Hochhaus et al. 2016b; Kantarjian et al. 2011; Larson et al. 2012; Saglio et al. 2010; Steegmann et al. 2016).

Patients with Ph−/*BCR-ABL1* + CML and those with unconfirmed Ph status at screening and no Ph+ metaphases at later time points were not eligible for cytogenetic response analysis. Of the 983 patients evaluable, complete cytogenetic response rate was 67.3% (*n* = 662; 95% CI, 64.4–70.3%) by 6 months and 82.5% (*n* = 811; 95% CI, 80.1–84.9%) by 12 months (Hochhaus et al. 2016a). In the overall population in the ENEST1st study, 97% of the patients achieved *BCR-ABL1* ^IS^ ≤10% at 3 months (Hochhaus et al. 2016a), a molecular target which is recommended for the achievement of better long-term outcomes (Baccarani et al. 2013; NCCN 2016), as seen in prior studies (Hanfstein et al. 2012; Hughes et al. 2014; Jabbour et al. 2014; Marin et al. 2012). Based on the landmark analysis, greater proportion of patients with *BCR-ABL1* ^IS^ ≤1% at 3 months vs *BCR-ABL1* ^IS^ >1% at 3 months achieved MR^4^ (65.0 vs 24.1%) and MR^4.5^ (45.8 vs 14.5%) by 24 months (Hochhaus et al. 2016a). However, this conclusion cannot be drawn in Ph−/*BCR-ABL1* + patients due to lower patient count. In total, 12 of the 14 patients evaluable had *BCR-ABL1* ^IS^ ≤1% at 3 months, and only one patient had *BCR-ABL1* ^IS^ >1% at 3 months; by 24 months, 58 and 41.7% of the patients with *BCR-ABL1* ^IS^ ≤1% at 3 months achieved MR^4^ and MR^4.5^, respectively.

The study was not designed to compare the two patient populations; also due to huge disparity between the numbers in each group, any meaningful comparisons cannot be drawn.

In conclusion, baseline characteristics, risk scores, and MR rates were found to be similar between the Ph−/*BCR-ABL1* + and Ph+ subgroups, and nilotinib is active in this previously unexplored population. Adverse events observed in Ph−/*BCR-ABL1* + CML patients were also found to be similar to the ones observed in the Ph+ CML patients. In the background of similar molecular response and safety profiles seen in patients with Ph−/*BCR-ABL1* + CML when compared with the Ph+ CML population, this rare population subgroup benefits from nilotinib treatment in the same way as Ph+ patients.
